# Effect of Age on Bleeding on Probing (BOP) as an Indicator of Periodontal Inflammation in Patients Enrolled in Supportive Periodontal Therapy

**DOI:** 10.3290/j.ohpd.b898947

**Published:** 2021-01-26

**Authors:** Christoph A. Ramseier, Jean R. Fischer, Gino Fischer, Martin Schimmel

**Affiliations:** a Dentist, Department of Periodontology, School of Dental Medicine, University of Bern, Switzerland. Constructed the hypothesis, performed the analysis, edited the manuscript, contributed substantially to the discussion.; b Dentist, Department of Periodontology, School of Dental Medicine, University of Bern, Switzerland. Wrote the manuscript in partial fulfilment of requirements for a Dr. med. dent. degree.; c Professor, Department of Reconstructive Dentistry and Gerodontology, School of Dental Medicine, University of Bern, Switzerland. Proofread and edited the manuscript, contributed to the discussion.

**Keywords:** bleeding on probing, elderly, compliance, supportive periodontal therapy

## Abstract

**Purpose:**

To assess the effect of age on the mean percentage of bleeding on probing (BOP) during supportive periodontal therapy (SPT) in patients enrolled in SPT for at least 5 years.

**Materials and Methods:**

This study was performed as a retrospective analysis of data collected from SPT patients initially diagnosed with gingivitis or mild to severe periodontitis. Two groups of patients were selected: in group A, younger adults (age ≤ 35 years) were included while group B consisted of older SPT patients (age ≥ 65 years). BOP in the two groups was compared according to both disease severity and % compliance with SPT visits.

**Results:**

BOP in all patients (n = 236) was 19.2% (± 12.4). Group A (n = 110) presented mean BOP levels of 19.7% (± 11.8), while lower BOP levels of 18.7% (± 13.0) were found in group B (n = 126; p = 0.5272). Older patients demonstrating high % compliance had lower mean BOP levels (14.2% ± 9.5) than younger patients (18.0% ± 11.7; p = 0.0841). Similarly, BOP was lower in older patients with moderate (group B: 18.4% ± 12.1, group A: 19.3% ± 14.6, p = 0.0541) or severe periodontitis (group B: 22.4% ± 11.4, group A: 23.2% ± 14.0; p = 0.3440). In patients with moderate or severe periodontitis and higher % compliance with SPT, the mean BOP was statistically significantly lower in older patients than in younger patients (moderate: 14.4% ± 11.9 vs 19.4% ± 15.1, p < 0.0001; severe: 13.2% ± 11.1 vs 18.3% ± 17.5, p = 0.0170).

**Conclusion:**

Older patients enrolled in SPT may present lower levels of BOP. This finding should be considered when determining SPT intervals with elderly patients.

With life expectancy continuing to rise across the world, the proportion of elderly people suffering from chronic diseases is simultaneously steadily increasing.^29^ At the same time, people are increasingly retaining their natural teeth well into old age.^30^ Dental practice teams are seeing elderly patients more often with natural dentitions or fixed prosthetic teeth requiring professional dental care, even up to very old age. Treatment decision making for such care is generally guided by a risk assessment for the progression of periodontal disease: high-risk patients are scheduled for supportive periodontal therapy (SPT) at shorter intervals than in low-risk patients.

Bleeding on probing (BOP) has been utilized in SPT planning as a periodontal risk indicator for many years.^1,13,14,21^ Patients with a mean percentage of sites with BOP (BOP%) of ≤ 20% are periodontally stable, while those with a mean BOP% of ≥ 30% are considered to have an increased risk of periodontal disease progression.^11,20,25^ As demonstrated by Ramseier et al,^25^ however, periodontally stable smokers enrolled in supportive periodontal therapy have a mean BOP of less than 16%, while 23% is the calculated periodontal stability threshold for ex-smokers and non-smokers. Furthermore, several studies have demonstrated the important role of age-related changes in the periodontal immune response in the development of gingivitis.^6,10^ Both the innate and the acquired immune system of aging individuals undergo a number of changes which generally lead to immunosenescence, or a functional deterioration of the entire immune system with age.^7^ It was suggested that these processes mainly occur due to qualitative changes at the cellular level rather than to a general decrease in all immune functions.^7^ Additionally, inflamm-aging is an age-related change in the human immune system in which – contrary to immunosenescence – dynamic immune defense processes paradoxically lead to the increased activity of defense cells and their mediators.^3,16,27^ Inflamm-aging is characterized by the increased release of proinflammatory messengers and the increased susceptibility of elderly individuals to chronic diseases associated with it.^5^

Due to these alterations in the host response in the elderly, the question arises as to whether their BOP% levels differ from that of younger adults and, if so, whether their periodontal risk assessment and BOP% threshold values should be adjusted accordingly, as is the case for smokers. To date, little is known about possible associations of immunosenescence and inflamm-aging with periodontitis.^8,9^ If elderly patients were shown to have a lower BOP% than younger individuals, even in those with comparable severity of periodontal disease, clinical assessments of periodontal risk must take this difference into account. The present study was therefore designed to test for possible effects of age on bleeding on probing (BOP), an indicator of periodontal inflammation, by retrospective analysis of patient data collected at the MEDI Dental Hygiene School (MEDI-DH) in Bern, Switzerland in a previous study by Ramseier et al.^25^

## Materials and Methods

This retrospective longitudinal cohort study was based on the analysis of clinical and demographic data extracted from the files of patients treated at the MEDI Dental Hygiene School in Bern (MEDI-DH), Switzerland from 1985 to 2011.^24,25^

**Fig 1:**
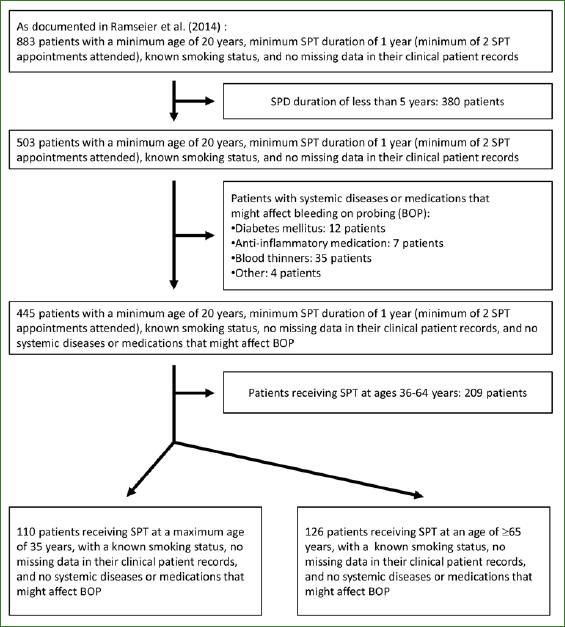
Patient selection diagram: Of 883 patients previously presented by Ramseier et al (2014), two subgroups of patients were identified with either a maximum age of 35 (n = 110) or a minimum age of 65 (n = 126). Patients in both subgroups returned for at least 5 years for supportive periodontal therapy (SPT).

Out of an overall population of 2213 patients treated at MEDI-DH during the specified period, a total of 445 were included in this study based on various eligibility criteria, as described by Ramseier et al.^25^ The inclusion criteria were: age ≥ 20 years and SPT enrollment for at least five years. Two subgroups of this sample were selected for further analysis: one contained younger SPT patients aged 20 to 35 years, and the other SPT patients aged 65 years or older (Fig 1). All patients in both subgroups had a known smoking status and medical status, no history of systemic disease, and were not taking any medications that might affect BOP.

Due to the retrospective nature of this analysis, no ethical approval from the Swiss Ethics Committee of the Canton of Bern, Switzerland was required. This analysis does not fall under the Swiss Research Act (HRA 810.30).

### Treatment Protocol at MEDI-DH

MEDI-DH used a standard screening procedure to evaluate all patients for all oral diseases on admission. If lesions on the oral mucosa were detected, the affected patients were referred to the dental clinics of the University of Bern for further diagnostic assessment and treatment. Patients with dental diseases or defective dental prostheses were either referred to their private dentists or treated by dentists at MEDI-DH. Periodontally healthy patients were managed at MEDI-DH according to in-house treatment standards. Periodontally diseased patients received initial periodontal therapy from dental hygiene students at MEDI-DH and were subsequently re-evaluated and referred for periodontal surgery, if necessary.

All patients treated at MEDI-DH received SPT. The necessary interval between SPT appointments was established at the end of each active periodontal therapy appointment and after each SPT appointment, and was later re-evaluated and adjusted as needed. The interval was determined based on the following criteria: 1. If the percentage of sites with BOP was > 20%, the interval was shortened by 1–2 months or to a minimum of 3 months; if the BOP was < 20%, the interval extended by 1-2 months up to a maximum of 12 months; 2. severity of periodontitis (sites with probing depths of 5 mm or more).

### Clinical History

The following demographic data, collected in the the first study,^25^ were extracted from the patient records for analysis in the present study: general health status, current medications and smoking status. The latter was categorized as follows: smoker (current smoker), former smoker (ex-smoker) and non-smoker (never smoked). The duration of active periodontal therapy, the first recall interval for supportive periodontal therapy after active periodontal therapy, and the total duration of SPT (in months) performed at MEDI-DH were studied as additional variables.

Retrospectively collected data from the patient records over the full duration of supportive periodontal therapy included the percentage of sites with bleeding on probing (BOP%), the number of natural teeth present, and the number of sites with periodontal probing depths (PPD) of 4 mm, 5 mm, 6 mm or ≥ 7 mm at six sites per tooth. In accordance with MEDI-DH policy, records were not kept on sites with probing depths of 0 to 3 mm.

### Statistical Analysis

All statistical analyses were performed using Predictive Analytics SoftWare (PASW, version 18.0.0, Polar Engineering and Consulting; Armonk, NY, USA). Mean, percentage, and standard error values were calculated by means of descriptive statistics. Multiple linear regression models and Fisher’s exact test were used to test for associations of demographic variables with mean BOP% and categorical variables. Student’s t-test and one-way ANOVA were used to test for statistical significance of differences between numerical variables within subgroups. p < 0.05 was defined as statistically significant.

## Results

### Demographic Analysis

The original patient population selected by Ramseier et al comprised 445 patients.^25^ Two subgroups of patients from that sample were included in the present study (Fig 1). The first (group A) consisted of 110 younger patients (age: 23-35 years) enrolled in SPT. The second (group B) comprised 126 older patients (age: ≥ 65 years) enrolled in SPT. The demographic characteristics of the sample are summarised in Table 1.

**Table 1:** Demographic data on 236 patients enrolled in supportive periodontal therapy (SPT), divided into two groups as follows: n = 110 patients aged 23 to 35 years and n = 126 patients aged 65 to 90 years

	All patients			23- to 35-year-olds (Group A)			65- to 90-year-olds (Group B)			p-value, difference between groups A and B (not adjusted)
	n = 236 patients (100%)			n = 110 patients (46.6%)			n = 126 patients (53.4%)			
	Mean	± SD	Range	Mean	± SD	Range	Mean	± SD	Range	p-value
Age (years) at start of treatment	43.6	± 15.7	20 – 81	28.2	± 3.3	20 – 35	57.0	± 8.0	39 – 81	<0.0001
Age (years) during SPT	51.8	± 18.7	23 – 90	32.1	± 2.2	23 – 35	68.9	± 3.9	65 – 90	<0.0001
Sex (female)	136	57.6%	n.a.	65	59.1%	n.a.	71	56.3%	n.a.	0.6722
Non-smoker	127	53.8%	n.a.	54	22.9%	n.a.	73	30.9%	n.a.	0.1919†
Smoker	50	21.2%	n.a.	37	15.7%	n.a.	13	5.5%	n.a.	<0.0001†
Ex-smoker	59	25%	n.a.	19	8.1%	n.a.	40	16.9%	n.a.	0.0109†
Duration of periodontal therapy (months)	5.5	± 4.6	0.0 – 34.1	5.1	± 4.6	0.0 – 29.5	5.9	± 4.5	0.3 – 34.1	0.2006
No. of SPT appointments	9.8	± 8.1	1 – 43	6.8	± 4.3	1 – 18	12.3	± 9.6	1 – 43	<0.0001
SPT interval (months)	6.0	± 2.4	3 – 12	6.6	± 2.5	3 – 12	5.5	± 2.1	3 – 12	0.0004
SPT duration (years)	9.6	± 6.6	0 – 25.0	4.5	± 3.1	0 – 11.8	13.9	± 5.6	5.2 – 25.0	<0.0001
% compliance with SPT	68.1	± 20.3	10.8 – 115.4	65.1	± 20.9	13.5 – 111.7	70.7	± 19.4	10.8 – 115.4	0.0358

SD: standard deviation; SPT: supportive periodontal therapy; % compliance: percentage of scheduled SPT appointments attended; †: Fisher’s exact test; n.a.: not applicable, range (minimum-maximum).

The mean age of the overall sample of 236 patients before the start of treatment was 43.6 (± 15.7) years, with a range of 20 to 81 years (minimum to maximum). Before the start of treatment, the younger patients (group A) had a mean age of 28.2 (± 3.3) years, with ages ranging from 20 to 35 years, whereas the older patients (group B) had a mean age of 57.0 (± 3.3) years within a range of 39 to 81 years. During participation in the SPT program, the mean age was 51.8 (± 18.7) years for the overall study population, 32.1 (± 2.2) years for group A, and 68.9 (± 3.9) years for group B.

The two groups had comparable gender distributions (p = 0.6722), and their smoking status category results (21.2% smokers, 25% ex-smokers and 53.8% non-smokers) were consistent with the general smoking prevalence rates among the Swiss population in about 2010. Groups A and B contained comparable percentages of non-smokers (p = 0.1919) but, as expected, the younger group had a higher proportion of active smokers (p < 0.0001), and the older group had a higher percentage of ex-smokers (p = 0.0109) (Table 1).

The duration of periodontal therapy did not differ statistically significantly between the two groups (5.1± 4.6 months in group A vs 5.9± 4.5 months in group B; p = 0.2006), but a statistically significant difference in SPT characteristics was observed: as expected, the older patients in group B had more SPT appointments (p < 0.0001), shorter SPT intervals (p = 0.0004), and more years of SPT overall (p < 0.0001). The rate of attendance of scheduled SPT appointments (% compliance) was also statistically significantly better (p = 0.0358) in group B than in group A (Table 1).

The classification applied by Ramseier et al^25^ was used to divide the pre-treatment severity of periodontal disease into the following three categories for further analysis: P1: gingivitis or mild periodontal disease with periodontal probing depths (PPDs) of 4 mm or less; P2: moderate periodontal disease with PPDs of 4 mm and more (but < 10) sites with PPDs of 6 mm or greater; P3: severe periodontal disease with probing depths of 4 mm or more and 10 or more sites with PPDs of 6 mm or deeper.

### Multiple Linear Regression Analysis

Multiple linear regression analysis revealed that two variables had a statistically significant effect on the mean BOP% in the 236 SPT patients included in the analysis (Table 2): % compliance, or rate of SPT attendance (p = 0.0132), and pre-treatment severity of periodontal disease (p = 0.0237), as defined using the three categories described above (P1, P2 and P3). The regression coefficient for the latter variable was 3.289 and was thus weighted most during further analysis.

**Table 2:** Multiple linear regression analysis of associations between mean BOP% and possible confounders weighted according to the duration of supportive periodontal therapy (SPT) in 236 SPT patients

	Regression coefficient	Standard error	Beta	T	95% confidence interval	p-value
Constant variable	37.675	6.731		5.598	24.412 – 50.938	<0.0001*
Age at start of treatment	-0.188	0.197	-0.218	-0.955	-0.576 – 0.200	0.3404
Age during SPT	0.098	0.156	0.142	0.627	-0.210 – 0.405	0.5311
Gender	-2.671	1.638	-0.104	-1.630	-5.900 – 0.557	0.1044
Number of SPT appointments	-0.638	0.592	-0.108	-1.079	-1.805 – 0.528	0.2985
Interval between SPT appointments	-0.015	0.006	-0.165	-2.547	-0.026 – -0.003	0.2819
Severity of disease	3.289	1.444	0.167	2.278	0.444 – 6.133	0.0237*
% compliance with SPT	-0.151	0.061	-0.228	-2.498	-0.270 – -0.032	0.0132*
Smoking status	-0.817	1.047	-0.051	-0.780	-2.881 – 1.248	0.4365
Periodontal stability	-0.117	1.978	-0.004	-0.059	-4.015 – 3.782	0.9529

SPT: supportive periodontal therapy; % compliance (percentage of scheduled SPT appointments attended); BOP%: percentage of sites with bleeding on probing; * statistically significant difference.

### Rate of SPT Attendance (% Compliance)

As described by Ramseier et al,^24^ % compliance was defined as the rate of attendance of scheduled SPT appointments, i.e. the percentage of SPT appointments attended. High compliance was defined as % compliance ≥ 68.1%, and low compliance as % compliance < 68.1%.

As shown in Table 3, the mean BOP% for the overall population of 236 patients was 19.2% (± 12.4). The younger group A had a mean BOP of 19.7% (± 11.8). That of the older group B was slightly lower (18.7% ± 13.0), but the difference was not statistically significant (p = 0.5272).

**Table 3:** Mean BOP by % compliance (high or low) with supportive periodontal therapy (SPT) in 236 patients enrolled in SPT

	All patients		20- to 35-year-olds (Group A)		≥ 65-year-olds (Group B)		p-value, difference between A and B (not adjusted)
	n = 236 (100%)		n = 110 patients (46.6%)		n = 126 patients (53.4%)		
	Patients (n)	BOP% (± SD)	n	BOP% (± SD)	n	BOP% (± SD)	p-value
All patients	236	19.2 (± 12.4)	110	19.7 (± 11.8)	126	18.7 (± 13.0)	0.5272
High % compliance (≥ 68.1%)	101	16.5 (± 10.6)	37	18.0 (± 11.7)	64	14.2 (± 9.5)	0.0841
Low % compliance (< 68.1%)	135	22.7 (± 13.6)	73	20.3 (± 11.7)	62	25.3 (± 15.1)	0.2363

SD: standard deviation; % compliance (percentage of scheduled SPT appointments attended); BOP: percentage of sites with bleeding on probing.

Older patients with high % compliance had a lower mean BOP (14.2% ± 9.5; n = 64) than younger patients with high % compliance (18.0% ± 11.7, n = 37), which was not quite statistically significant (p = 0.0841) (Table 3). In subjects with low % compliance, there was no statistically significant difference in mean BOP% between those in age groups A and B.

### Pre-treatment Severity of Periodontal Disease

As shown in Table 4, the mean BOP% in P1 patients (n = 34) with gingivitis or mild periodontitis (P) was 12.5% (± 7.0). Mean BOP% values were much higher in subjects with higher levels of disease severity: 20.3% (± 11.9) in P2 patients (n = 131) with moderate periodontitis, and 20.3% (± 14.4) in P3 patients (n = 71) with severe periodontitis. Furthermore, the mean percentage of sites with BOP was lower in older P2 and P3 patients than in younger P2 and P3 patients, but the difference was not statistically significant: 18.4% (± 12.1) and 19.3% (± 14.6) in group B vs 22.4% (± 11.4) and 23.2% (± 14.0) in group A, respectively (Table 4).

**Table 4:** Mean BOP by periodontal disease severity in 236 patients enrolled in supportive periodontal therapy (SPT)

	All patients		20- to 35-year-olds (Group A)		≥ 65-year-olds (Group B)		p-value, difference between A and B (not adjusted)
	n = 236 patients (100%)		n = 110 patients (46.6%)		n = 126 patients (53.4%)		
	Patients (n)	BOP (± SD)	n	BOP (± SD)	n	BOP (± SD)	p-value
All severity categories	236	19.2 (± 12.4)	110	19.7 (± 11.8)	126	18.7 (± 13.0)	0.5272
P1: Gingivitis or mild CP	34	12.5 (± 7.0)	29	11.9 (± 7.1)	5	16.2 (± 4.6)	0.2055
P2: Moderate CP	131	20.3 (± 11.9)	64	22.4 (± 11.4)	67	18.4 (± 12.1)	0.0541
P3: Severe CP	71	20.3(± 14.4)	17	23.2 (± 14.0)	54	19.3 (± 14.6)	0.3440

CP: chronic periodontitis; SD: standard deviation; BOP: percentage of sites with bleeding on probing.

### Mean BOP% by Percent Compliance and Severity of Periodontal Disease

As shown in Table 5, our analysis of the data at the level of the individual SPT appointments showed that older patients (group B) with moderate (14.4% ± 11.9) and severe periodontitis (13.2% ± 11.1) had significantly lower mean BOP% values than their younger counterparts (p < 0.0001 and p = 0.0170, respectively). No other statistically significant differences between the groups were detected in further comparisons.

**Table 5:** Mean BOP by % compliance and severity of periodontal disease in 236 patients enrolled in supportive periodontal therapy (SPT)

	All patients		20- to 35-year-olds (Group A)		≥ 65-year-olds (Group B)		p-value, difference between A and B (not adjusted)
	N = 236 patients (100%)		N = 110 patients (46.6%)		N = 126 patients (53.4%)		
	Number of SPT appts	BOP (± SD)	Number of SPT appts	BOP (± SD)	Number of SPT appts	BOP (± SD)	p-value
High % compliance (≥ 68.1%)	1,180	16.5 (± 10.6)	317	18.0 (± 11.7)	863	14.2 (± 9.5)	0.0841
P1: Gingivitis or mild CP	151	15.8 (± 12.4)	113	15.0 (± 13.1)	38	18.3 (± 10.0)	0.1575
P2: Moderate CP	516	16.1 (± 13.3)	173	19.4 (± 15.1)	343	14.4 (± 11.9)	<0.0001*
P3: Severe CP	513	13.5 (± 11.6)	31	18.3 (± 17.5)	482	13.2 (± 11.1)	0.0170*
Low % compliance (< 68.1%)	1,124	22.7 (± 13.6)	434	20.3 (± 11.7)	690	25.3 (± 15.1)	0.2363
P1: Gingivitis or mild CP	109	11.6 (± 12.0)	101	11.8 (± 12.2)	8	11.1 (± 9.1)	0.8643
P2: Moderate CP	621	23.0 (± 11.6)	267	23.6 (± 18.4)	354	22.6 (± 17.0)	0.4975
P3: Severe CP	394	22.0 (± 17.0)	66	20.6 (± 16.9)	328	22.3 (± 17.1)	0.3764

CP: chronic periodontitis; SD: standard deviation; BOP: percentage of sites with bleeding on probing; appts: appointments; *statistically significant difference.

## Discussion

The findings of this study show that both the severity of periodontal disease and the % compliance with SPT attendance have a significant effect on the mean percentage of sites with BOP, independent of the age of the patient enrolled in supportive periodontal therapy. The pre-treatment severity of periodontal disease had the greatest effect on the mean BOP%. A particular finding of this retrospective study is that among the subjects with moderate to severe periodontitis who maintained high % compliance with SPT, older patients ≥ 65 years of age had statistically significantly lower BOP% than their younger counterparts. Among patients with low % compliance, there was no statistically significant difference in BOP% between the older and younger groups.

Schürch and Lang^26^ analysed the periodontal status of the Swiss population in the scope of an epidemiological study in 2004. They were particularly interested in potential associations between patient age and indicators such as the Gingival Index (GI), as described by Löe and Silness (1963).^19^ The mean GI score for their sample of 1224 subjects was 1.32 (± 0.50). The difference between young and old subjects was statistically significant. The mean GI score in young adults aged 20 to 29 years was 1.17 (± 0.34) compared to elevated levels of 1.51 (± 0.38) in elderly subjects aged 70 to 79 years and 1.64 (± 0.50) in patients ≥ 80 years of age. In the present study, we detected an overall but slight tendency towards an age-related increase in BOP in patients with mild periodontitis or gingivitis, but the difference was not statistically significant. Our findings, however, demonstrate that the mean percentage of sites with BOP decreased with age in the pre-treatment severity categories of moderate and severe periodontitis. Therefore, even though a slight increase of the gingival index in the elderly was seen in our sample, the comparability of our results with those of the study by Schürch and Lang^26^ may be limited, possibly since their patients were not enrolled in SPT and they did not use BOP or make comparisons within the groups ‘disease severity’, ‘smoking status’ or ‘compliance’.

Trombelli et al^28^ conducted a study in Italy to test for age-dependent responses to nonsurgical periodontal therapy. Fifty-seven young adults with a mean age of 34.7 (± 4.4) years and sixty older adults with a mean age of 58.9 (± 5.3) years were included in their analysis. Variables studied included BOP% before and after periodontal therapy. Before treatment, the mean BOP% was 30.9% in the younger group compared to 27.5% in the older group. The same pattern was seen following periodontal therapy: the younger patients had a mean BOP% of 16.0% compared to 15.6% in the older patients. Similar to our study, their older subjects also had lower BOP% values both before and after treatment. Based on the fact that the patients in the study by Trombelli et al^28^ were receiving periodontal therapy, one may assume that the majority of their patients had mild or severe periodontitis. Thus, their results overlap with those in our sample while confirming our hypothesis that BOP may be reduced in the elderly.

In a much earlier study, Holm-Pedersen et al^10^ examined a test population for age-related differences in gingival inflammation and concluded that the older subjects had higher GI scores. However, as that study was based on a model of experimental gingivitis using subjects without periodontitis at baseline, no direct comparisons can be drawn between their results and ours. However, it is interesting to note that Holm-Pedersen et al^10^ detected virtually no difference between the age groups at baseline, before the induction of experimental gingivitis. The difference only occurred during the experimental phase when the subjects were required to stop all home oral hygiene measures for 21 days. Apart from the GI, Holm-Pedersen et al^10^ also studied the plaque index (PI) and found that their older subjects had much higher PI values than their younger counterparts. Moreover, the age-related difference in the PI was significantly greater than that for the GI. Consequently, the GI values in their older subjects increased only slightly despite a massive increase in plaque accumulation possibly confirming our hypothesis that BOP in the elderly may be reduced due to overall changes of the host response. Holm-Pedersen et al^10^ also examined the production of sulcus fluid during experimental gingivitis. Here, too, their older subjects showed much higher levels of sulcus exudate production during the development of experimental gingivitis. The increased sulcus fluid production observed in older subjects might be a result of inflamm-aging, while the slight increase in GI in combination with a sharp increase in PI may be a sign of immunosenescence.

Bleeding on probing (BOP) served as a primary variable of interest in the present study as an indicator of gingival inflammation. As described by Lang et al,^14^ BOP is mainly caused by the pressure of the periodontal probe at the inflamed bottom of the periodontal pocket. Bacteria in the oral biofilm activate the immune system defence cells of the marginal periodontium. Local blood flow is then increased to enable these cells to better reach the source of infection via the bloodstream. This increase in blood flow is the reason why bleeding at a BOP-positive site can be interpreted as a sign of gingival inflammation in clinical diagnostics.^22^

Neutrophil granulocytes – the most abundant type of white blood cell in the immune system – form an important part of the innate immune system. Neutrophils are formed in the bone marrow and serve as a first line of defense against invading pathogens. They are able to envelop microorganisms by phagocytosis and attract other defense cells to a site of infection or inflammation through chemotaxis, ie, chemical signals.^12^ Age is associated with various changes or impairments of many neutrophil functions, including chemotaxis and susceptibility to induced apoptosis as well as the phagocytosis of microorganisms and the production of reactive oxygen species.^9^ Age causes a decline in all of these functions except induced apoptosis, which increases due to a decrease in the release of anti-apoptotic signals, such as granulocyte macrophage-colony stimulating factor (GM-CSF).^2^ Many possible reasons for these changes in function have been discussed. Inflamm-aging (low-grade inflammation) is only one of them. Neutrophil hypo-responsiveness due to age-related changes in stem cell properties may also play a role. Other important changes occur at the molecular level over the course of aging: intracellular and transmembrane signal cascades are impaired by various activation and inhibition processes. Changes in membrane composition (lipid rafts), reduced recruitment of key membrane receptors such as Toll-like receptors, and age-related changes in intracellular signal cascades have been proposed as possible reasons for this.^4,15^

Another change involves prostaglandin E2 (PGE-2), the production of which increases with age. PGE-2 plays an important role in inflammatory resorption of the periodontium.^23^ Increased periodontal bone loss in older individuals is generally accompanied by the increased expression of proinflammatory cytokines. One group of researchers found that ageing mice express significantly increased levels of interleukin-1-beta (IL-1β) and tumour necrosis factor-alpha (TNF-α) – two key cytokine mediators of the destructive bone resorption associated with periodontitis.^23^ Another murine study by Liang et al^17,18^ revealed the presence of age-related alterations in the expression of 6 out of 15 gingival immune receptors tested. Those authors assumed that some of these receptors contribute to increased periodontal inflammation because they are involved in amplification of the host inflammatory response. Hajishengallis et al^8,9^ also observed the upregulation of other types of immune receptors with age.

The data collected by the MEDI-DH in Bern provide an extensive set of information about periodontal conditions in patients who have received long-term professional dental hygiene care for at least 5 years. As periodontal probing depths were measured by dental hygiene students at MEDI-DH, clinical data collection could be subject to measurement inaccuracies due to the different levels of clinical experience of the examiners. However, all measurements were checked and corrected as needed by instructors with many years of clinical experience.

Because of the retrospective design of the study, the study was limited to data that had been collected before the specific research questions, criteria and groups investigated in this study had been defined. The large sample size, however, enabled division of the population into relatively large subgroups in spite of age selection. Furthermore, the fact that the patients in the selected subgroups were not on any medications that might affect BOP as an indicator of gingival inflammation might limit the clinical relevance of this study because a large number of elderly patients take such medications. Thus, the results of this study can only be directly applied to patients receiving professional SPT who are not taking any medications that affect BOP.

## Conclusion

It is a fact that the longevity of the world’s population is increasing. The consequence of this for routine clinical practice is that the number of elderly patients seen in dental practices will continue to rise in the future. Elderly patients today can also retain their natural teeth (and periodontium) longer than ever before, especially in the Western world. Strategies to boost research in our field should include prospective cohort studies that focus on residual pockets in the elderly. When conducting such studies, it is important to make clear distinctions between systemically healthy elderly subjects and those with general diseases that may affect the periodontium and its inflammatory activity.

While aspects of both inflamm-aging and immunosenescence are expected in the elderly, in the present study, the clinical expression of the latter in the periodontium was found in patients with higher % compliance and initial diagnosis of moderate to severe periodontitis. Therefore, while mean BOP levels of < 20% in younger adults may still reflect periodontal stability, in the elderly, lower BOP levels of < 15% may be needed. Consequently, patients exceeding this threshold should be scheduled for SPT at shorter intervals.
